# Psychedelics and the ‘inner healer’: Myth or mechanism?

**DOI:** 10.1177/02698811241239206

**Published:** 2024-04-12

**Authors:** Joseph Peill, Miriam Marguilho, David Erritzoe, Tommaso Barba, Kyle T Greenway, Fernando Rosas, Christopher Timmermann, Robin Carhart-Harris

**Affiliations:** 1 Division of Psychiatry, Department of Brain Sciences, Centre for Psychedelic Research, Imperial College London, London, UK; 2 Division of Psychiatry, Lisbon Psychiatric Hospital Centre, Lisbon, Portugal; 3 Faculty of Medicine, Department of Psychiatry, McGill University, Ludmer Research and Training Building, Montréal, QC, Canada; 4 Lady Davis Institute, Jewish General Hospital, Montréal, QC, Canada; 5 Centre for Complexity Science, Imperial College London, London, UK; 6 Department of Informatics, University of Sussex, Brighton, UK; 7 Centre for Eudaimonia and Human Flourishing, University of Oxford, Oxford, UK; 8 Departments of Neurology and Psychiatry, Carhart-Harris Lab, University of California San Francisco, San Francisco, CA, USA

**Keywords:** Healing, Telos, psychedelic, automatic

## Abstract

**Background:**

Reference to an intrinsic healing mechanism or an ‘inner healer’ is commonplace amongst psychedelic drug-using cultures. The ‘inner healer’ refers to the belief that psychedelic compounds, plants or concoctions have an intrinsically regenerative action on the mind and brain, analogous to intrinsic healing mechanisms within the physical body, for example, after sickness or injury.

**Aims:**

Here, we sought to test and critique this idea by devising a single subjective rating item pertaining to perceived ‘inner healing’ effects.

**Methods:**

The item was issued to 59 patients after a single high (25 mg, *n* = 30) or ‘placebo’ (1 mg, *n* = 29) dose of psilocybin in a double-blind randomised controlled trial of psilocybin for depression.

**Results:**

Inner healer scores were higher after the high versus placebo dose of psilocybin (*t* = 3.88, *p* < 0.001). Within the high-dose sub-sample only, inner healer scores predicted improved depressive symptomatology at 2 weeks post-dosing.

**Conclusions:**

The principle of activating inner healing mechanisms via psychedelics is scientifically nascent; however, this study takes a positivist and pragmatic step forward, asking whether it warrants further examination.

## Introduction

Regeneration or ‘intrinsic healing’ refers to the capacity of living systems to restore or recover after injury or sickness. That is, ‘healing’ refers to the recovery, repair or restoration of health, and ‘intrinsic’ implies that the healing process has its causal origin within the living organism itself, as opposed to relying on causation via an external intervention.

There is a long history of interest in neural regeneration (Stahnisch, 2021) and now renewed interest in regenerative interventions for neurodegenerative disorders and nervous system injury (Huang et al., 2021). When applied to mental health, the principle postulates implicit processes that have an intrinsic directionality or ‘teleology’ to them – that is, they move towards an end goal which, in the present context, may be (non-exclusively) healing, recovery or ‘wholeness’ (Grof, 2012; Vaid & Walker, 2022). The implicit, dynamic processes would be examples of ‘entelechy’ – meaning they are psychological and biological processes that move towards the goal of healing.

Analogies are often made between intrinsic psychological and physical healing, as well as self-regulating processes elsewhere in living systems (Varela et al., 1974). The ‘inner healing’ theme can be found in traditions and cultures across the world and throughout history (Campbell, 2008); as well as in many integrative health and wellness, spiritual and religious practices – including yoga, psychotherapy, breathwork, meditation and prayer. Activating regenerative mechanisms also bears relevance to ‘hormetic’ stress (Epel, 2020) and the ‘Jarisch-Herxheimer reaction’ or ‘healing crisis’ (Bryceson, 1976).

Inner or intrinsic healing is a popular theme in psychedelic therapy. Indeed, ‘healing’ is arguably the oldest and most preserved intention for psychedelic plant, fungi or drug use, alongside ritual and psychological insight (Hofmann, 2013; Muraresku, 2020). The historicity and contemporaneity of healing as a theme within psychedelic medicine and use culture could be viewed as suggestive of a common intuition regarding the role of regeneration in their action. Moreover, the inner healer theme is not only commonplace in the psychedelic therapy ‘underground’ but also in psychedelic therapy trials.

For example, drawing inspiration from the transpersonal psychology of Grof (2008), the Multidisciplinary Association for Psychedelic Studies 3,4 Methylenedioxymethamphetamine (MDMA)-assisted psychotherapeutic model for post-traumatic stress disorder encourages patients to ‘listen’ or ‘let go’ to the ‘inner healer’ (Mithoefer et al., 2008). Indeed, the term ‘holotropic’ from Stanislav Grof’s ‘holotropic breathwork’ is intended to denote a process of moving towards ‘wholeness’, where ‘holos’ is from the Greek word ‘holos’, for ‘whole’ – and ‘trepein’ means ‘moving towards’ or ‘directed towards’. Thus, teleological philosophies and mechanisms are implicit in major psychedelic therapy training and approaches.

In the present work, we begin to ask whether ‘inner healing’ refers to a legitimate mechanistic component of psychedelic therapy, or is an example of mythmaking exploiting the known role of priming and expectation in shaping psychotherapy outcomes (Cuijpers et al., 2019) – particularly given the suggestibility-enhancing properties of psychedelics (Carhart-Harris et al., 2015). The present article is intended to draw attention to this issue as a matter for further thought, discussion, debate and empirical research. Furthermore, we offer preliminary data on a single item pertaining to the inner healer taken from a recent double-blind randomised controlled trial (DB-RCT) of psilocybin versus escitalopram for major depressive disorder (MDD) (Carhart-Harris et al., 2021).

One might perceive an overlap between the notion of ‘inner healing’ and certain psychological constructs that have been formalised, measured and found to be relevant to psychedelic therapy, including emotional breakthrough, release or catharsis (Roseman et al., 2019), psychological insight (Davis et al., 2021; Peill et al., 2022) and psychological connectedness (Carhart-Harris et al., 2018a; Watts et al., 2022). The construct of the ‘mystical-type experience’ (Griffiths et al., 2011; MacLean et al., 2011) has received much attention in psychedelic research and is also relevant. For example, one item in the commonly used mystical experience questionnaire (MEQ) reads ‘Gain of insightful knowledge experienced at an intuitive level’. However, the MEQ has been critiqued for invoking metaphysical transcendentalism (Taves, 2020), a philosophical position that postulates the existence of phenomena that transcend definition or measurement and therefore scientific praxis. Transcendentalism is common when constructs remain vague and ill-defined – as is the case with ‘the inner healer’. The scientific method is intended to deconstruct, test, and potentially validate such constructs.

In this paper, we investigate whether the inner healer construct references a ‘true’ and important phenomenon deserving of greater empirical investigation. Our approach is positivist and secular. We are specifically interested in whether the inner healer construct has mechanistic and predictive validity. Thus, we see the present work as a first step towards testing the validity of the inner healer construct. As with the ‘mystical-type experience’ (Griffiths et al., 2011), we recognise that the outcome of this process may ultimately entail a reframing of the original construct that is, as its original vagueness and abstraction breaks down into more tangible, testable phenomena, that is, the process of ‘demystification’. In the context of the inner healer, what may result, is a deeper understanding of the phenomenon that brings it closer to the notion of regeneration in physical health and medicine.

As a pragmatic – though admittedly limited – step forward, here we have created a single subjective rating item pertaining to the concept of the ‘inner healer’. This item was issued to participants after dosing sessions in a DB-RCT of psilocybin versus escitalopram for depression (Carhart-Harris et al., 2021). The item was administered to participants at the end of the first dosing day involving either 1 mg psilocybin, a ‘placebo’ control condition, or 25 mg psilocybin, the active condition.

We hypothesised that (1) there would be higher scores on the inner healer item for the 25 mg psilocybin experience versus the 1 mg experience and (2) that scores on this item for the 25 mg psilocybin condition (and not the 1 mg condition) would be predictive of clinical outcomes at one of the trial’s endpoints (i.e. 2 weeks after first treatment inception).

Positive findings would be interpreted as preliminary evidence for the predictive validity of the inner healer construct in relation to psychedelic therapy, thereby motivating further research on the topic. Moreover, we also sought to examine whether baseline suggestibility and expectancy could predict scores on the inner healer item. A negative result here could be interpreted as implying that the inner healer phenomenon is *not* a mere product of psychological priming but rather has a more basic biological underpinning. We also sought to explore how the inner healer item relates to the mystical experience, emotional breakthroughs, challenging experiences and altered states of consciousness (ASC) rated in relation to the acute phase of the psychedelic experience. This was done to determine evidence for the convergent validity or overlap (vs the discriminant validity or orthogonality) between the inner healer construct, and better-validated constructs/measures that are currently used in the context of psychedelic research.

## Methods

### Ethics

This study was approved by the Imperial College Research Governance and Integrity Team and Brent HS Research Ethics Committee the U.K. Medicines and Healthcare Products Regulatory Agency, the Health Research Authority, the Imperial College London Joint Research Compliance and General Data Protection Regulation Offices, and the risk assessment and trial management review board at the trial site – the National Institute for Health Research and Imperial Clinical Research Facility. All participants gave written informed consent before admission to the study.

### Psychometrics

#### The inner healer

The *inner healer item* is a single 5-point Likert scale item that is designed to be completed after the psilocybin dosing experience. The item was issued at the end of dosing days, in the period known to coincide with absent or negligible subjective drug effects, that is, the same timepoint as other acute measures following the experience. We use ‘negligible’ here in a manner consistent with its dictionary definition, which reads: ‘*so small or unimportant as to be not worth considering*’. Thus, the participant can be assumed to have returned to normal waking consciousness when completing these measures. The inner healer item was intended to capture how the participants felt in relation to their recent experience. The scale is answered in single unit integers from −2 to +2, where a score of −2 equals ‘strongly disagree’, a score of +2 equals ‘strongly agree’ and a score of 0 equals ‘neither agree nor disagree’. The inner healer item reads as the following:

I felt like my body/mind/brain was healing itself, automatically/naturally/by itself.

#### Beck depression inventory

The beck depression inventory (BDI) is a validated, 21-item self-assessment of depressive symptoms scored from 0 to 3, originally created by Beck et al. (1996). The scale covers various facets of depressive symptomatology including sleep, mood, appetite, concentration, guilt, interest, libido, irritability, agitation, self-dislike and fatigue (Beck et al., 1996). The scale is useful for screening depression in various phases of the disease (Viinamäki et al., 2004); it has a high Cronbach’s alpha (*a* = 0.904) (Biracyaza et al., 2021) and has shown good convergent validity for example, with the Montgomery-Asberg Depression Rated Scale; a clinician-rated scale for depressive symptoms (Englbrecht et al., 2017). Here, we used the BDI version 1A. Note: we failed to attain BDI scores at 2 weeks for just one participant. The BDI was chosen to assess the predictive validity of the inner healer item due to its apparent mechanistic sensitivity and validity in relation to psilocybin (Daws et al., 2022). We chose not to use the Quick Inventory for Depressive Symptomatology - Self Rated assessment 16 (QIDS-SR16) due to problems that have been identified with the scale, as described in detail here (Weiss et al., 2023).

#### Emotional breakthrough inventory

A six-question survey developed by Roseman et al. (2019) measures acute emotional breakthroughs using the Visual Analogue Scale (VAS) with scores ranging from 0 (‘No, not more than usual’) to 100 (‘Yes, entirely or completely’). The emotional breakthrough inventory (EBI) score, correlated with long-term well-being (2 weeks), demonstrates predictive validity and exhibits high internal consistency (Cronbach’s α = 0.932).

### Mystical experience questionnaire

The revised 30-item MEQ evaluates acute mystical-type experiences using a 5-point Likert-type scale, categorising responses into four subscales: ‘Mystical’, ‘Positive Mood’, ‘Transcendence of Time and Space’ and ‘Ineffability’ (MacLean et al., 2011). With high internal consistency (Cronbach’s α = 0.933), the MEQ exhibits strong predictive validity for long-term psychological measures like personal meaning (Garcia-Romeu et al., 2019; Griffiths et al., 2008).

#### Challenging experience questionnaire

Developed by Barrett et al. (2016), the challenging experience questionnaire (CEQ) assesses challenging events during psychedelic experiences using a 26-item, 5-point Likert-type scale. It includes seven subscales: Fear, Paranoia, Insanity, Physical Distress, Isolation, Death and Grief. Subscale internal consistency ranged from Cronbach’s α = 0.65 to 0.89, while overall internal validity for the total scale was later reported as excellent (Cronbach’s α = 0.95; Davis et al., 2021).

#### ASC questionnaire

Taken from Studerus et al. (2010), we used the 11-D ASC which incorporates 11 validated subscales: insightfulness, disembodiment, complex imagery, elementary imagery, spiritual experience, audio-visual synesthesia, changed meaning of percepts, impaired control of cognition, blissful state experience of unity and anxiety. Demonstrating good convergent and discriminant validity, this scale is a factor analysis of items from the original ASC (Dittrich, 1998).

#### General intensity of drug effects

The general intensity of subjective drug effects was assessed using a standard 100-increment VAS. As with all the acute subjective rating scales, this VAS item was performed once the acute drug effects had subsided to a negligible level. The item reads as follows:

Please rate the overall intensity of the drug effects when the effects were at their most intense.

The scale’s bottom anchor read ‘no effects’ and its top anchor read ‘extremely intense effects’.

## Study overview

This was a two-arm, between-subjects DB-RCT of investigational drug COMP360 (COMPASS Pathways proprietary synthetic psilocybin) with psilocybin therapy versus a 6-week course of the selective serotonin reuptake inhibitor, escitalopram, administered alongside matching psychological support for MDD. For a detailed account of the study design, please see the original study report (Carhart-Harris et al., 2021). Recruitment of participants was through word-of-mouth and online advertisements. In all, 59 patients with moderate-to-severe MDD were randomised to one of two arms: (1) two high-doses (25 mg) of psilocybin, 3 weeks apart, plus daily placebo after the first psilocybin dosing session or (2) two placebo-doses (1 mg) of psilocybin, 3 weeks apart, plus daily escitalopram for 6 weeks, that is, 10 mg escitalopram for the first 3 weeks and 20 mg escitalopram for the final 3 weeks. In the present publication, we focus exclusively on the 2-week window after the first dose of psilocybin. We do this as the inner healer scores were collected only after the first dose of psilocybin and not the second. The rationale was that the inner healer item was intended to refer to the 25 mg versus 1 mg psilocybin contrast and not the action of escitalopram per se.

### Statistical analyses and hypotheses

It was hypothesised that inner healer scores would be significantly higher in the 25 mg group versus the 1 mg psilocybin group. We also predicted that inner healer scores for the 25 mg experience would correlate with subsequent improvements in depressive symptomatology. T-tests and Pearson correlations were performed to test these hypotheses with a standard alpha of <0.05. One-tailed tests are justified by the unambiguous directionality of the prior hypotheses. All the statistical analyses were performed using SPSS V28 (IBM corporation, Armonk, New York, 2017), GraphPad Prism version 10.1.0 and RStudio: Integrated Development for R (RStudio Team, 2020), And RStudio, PBC (Boston, MA, USA).

## Results

A total of 59 participants took part in the study, with 30 participants in the psilocybin arm and 29 in the escitalopram arm. For detailed demographics, please see the original publication (Carhart-Harris et al., 2021).

**Figure 1.**
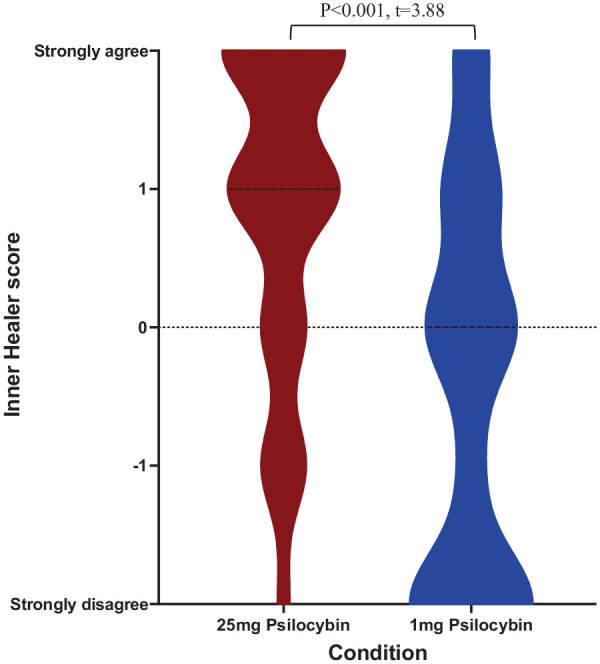
Distribution of inner healer scores after sessions with 25 mg versus 1 mg of psilocybin. 25 mg is known as a ‘high’ dose and 1 mg is assumed to be functionally inactive and therefore a ‘placebo’ dose. For display purposes, median values are shown. Scores range across all values for both groups. A significant difference between the groups was found (*p* < 0.001, *t* = 3.88).

As hypothesised, ratings for the inner healer item given by the 25 mg psilocybin group (*M* = 0.87, SD = 1.17) were significantly higher (*t* = 3.88, *p* < 0.001) than for the 1 mg group (*M* = −0.45, SD = 1.43). The upper 75th percentile modal score was 2 (25 mg group) and 1 (1 mg group). Lower 25th percentile modal scores were 0 (25 mg group) and −2 (1 mg group) (Figure 1).

## Inner healer and depression change

**Figure 2.**
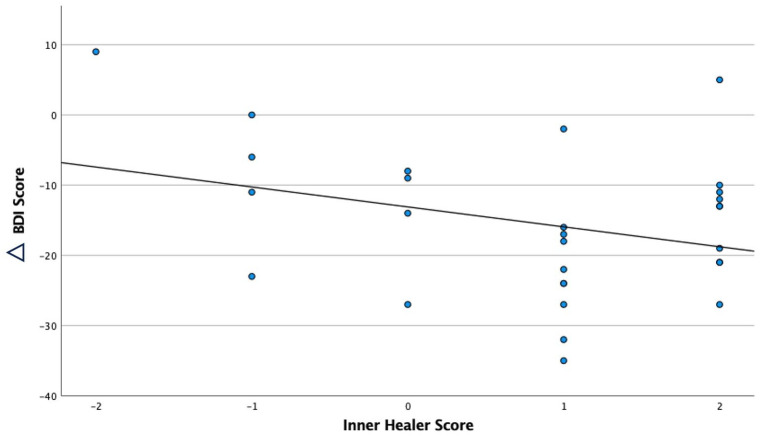
Correlation plot of inner healer (acute) and changes in Beck Depression Inventory (BDI) scores from baseline to 2 weeks post-single dose for the 25 mg psilocybin arm. The correlation was significant for BDI versus inner healer scores (*r* = −0.315, *p* < 0.05) at 2 weeks. Correlation is significant at the 0.05 level and was strengthened when general intensity was added as a covariate (−0.367, *p* = 0.028).

For the 25 mg condition, correlational analyses revealed a negative correlation between inner healer scores and changes in BDI scores from baseline to 2 weeks after the 25 mg session (*r* = −0.315, *p* = 0.048). When controlling for subjective drug intensity ratings, the negative correlation between the inner healer and BDI scores was preserved (*r* = −0.367, *p* = 0.028), Table 1, indicating that the inner healer effect was ‘over-and-above’ mere generic subjective drug effects, that is, it had some specificity in its relation to decrease depression scores. No correlation between the inner healer and BDI scores was apparent for the 1 mg condition at the 2-week timepoint (*r* = −0.110, *p* = 0.586). Moreover, controlling for pre-trial trait suggestibility and positive expectancy (for the received treatment) failed to explain the inner healer × depression change relationship, also supporting its potential causal importance (Figure 2).

**Table 1.** Correlations for 25 mg arm general subjective intensity and inner healer versus Beck Depression Inventory (BDI) change from baseline to 2 weeks post-dosing day 1 (DD1). The correlation was significant for BDI versus inner healer scores (−0.315, one-tailed) at 2 weeks.

Scale	Measure	General subjective intensity	Inner healer	Inner healer (controlling for intensity scores)
BDI (2 weeks post-DD1)	Pearson’s *r*	0.063	−0.315*	**−0.367** *
	*p* value (one-tailed)	0.372	**0.048**	**0.028**
	*N*	29	29	29

Bold values represent significant values. *Correlation is significant at below 0.05.

## Discussion

The present study sought to begin a process of testing the validity of the construct of the ‘inner healer’. To do this, we created a simple single subjective rating item to operationally define the phenomenon. In a randomised and blinded between-subjects design, scores on the item, which was rated at the end of dosing days, were compared between a high-dose psilocybin experience (25 mg) versus an inactive ‘placebo’ dose of psilocybin (1 mg) with matching psychological support. Secondarily, we examined whether scores on the inner healer item correlated with post-treatment changes in depressive symptom severity, and finally, we examined generic subjective intensity as a co-variate to inform inferences regarding the causal action of the inner healer phenomenon.

As predicted, scores on the inner healer item were significantly higher for those who received 25 mg psilocybin versus those who received the placebo dose; moreover, within the 25 mg group, the inner healer effect correlated with decreases in depressive symptom severity (as measured using the BDI) at a salient endpoint, 2 weeks after the intervention. Importantly, no such associations were found when examining the generic subjective intensity of effects produced by either dose. Moreover, when controlling for generic subjective intensity, the relationship between the inner healer and depression change was enhanced, implying that this relationship is independent of mere generic drug effects. Furthermore, pre-trial suggestibility and expectancy did not correlate with the inner healer effect, implying that the effect was not the product of mere psychological priming. These results support the inference that the inner healer construct indexes a substantive phenomenon causal to improvements in depressive symptomatology. We are left to speculate that it likely also has a substance neurobiological underpinning.

Supplementary analyses done on request as part of the review process examined relationships between the inner healer scores and scores for other aspects of the psychedelic experience, namely emotional breakthrough (EBI), mystical type experience (MEQ) and challenging experience (CEQ). This was done to further examine any statistical overlap between the inner healer phenomenon and other validated aspects of the psychedelic experience. As expected, we saw that when MEQ (but not EBI or CEQ) scores were added as a covariate in the inner healer versus BDI change models, the predictive relationship between the inner healer and decreased depression was diminished, implying that while the inner healer action may be independent of mere generic subjective drug effects, it likely overlaps in nature with the mystical-type experience constructs and phenomena. As these analyses were done on request, however, and due to the small sample size in this study, we are cautious about making strong inferences on these supplementary analyses.

This said these preliminary results do, tentatively, lend further support to the validity of the inner healer as a phenomenon of relevance to the acute and potential therapeutic action of high-dose psilocybin, suggesting at least some degree of orthogonality – or specificity – versus other features of the acute experience. We conclude from this that the inner healer construct does indeed warrant further investigation, particularly in relation to psychedelic medicine.

The inclusion of the present inner healer item in a DB-RCT of psilocybin versus escitalopram for depression was a small experiment within a larger one (Carhart-Harris et al., 2021). It was intended as a first attempt at operationally defining an abstract construct – that is, by examining how participants respond to a simple single-item index in a controlled experiment. Expansion of the inner healer item to a larger multi-item scale and study would be a logical next step to explore the phenomenon further (see NCT04505189 – clinicaltrials.gov in this regard), as would a focused qualitative analysis of the theme – as has been done successfully in the past with other psychedelic-relevant constructs such as a sense of ‘connectedness’ (Watts et al., 2017, 2022). In addition to assessing patients’ perception of the psychedelic having an intrinsically healing action, one might also examine patients’ perception of the locus of therapeutic action that is, do they attribute a healing action (1) directly to the drug, plant or fungi itself, (2) how it acts on the body and brain to alter its dynamics in a meaningful way, (3) how it alters the patient’s relationship to their therapists or ‘guides’ or (4) something else? We recognise that these possibilities are not mutually exclusive and may interact.

If future work does support the relevance of an (imagined, actual or both) inner healing action to psychedelics, it will be essential to better understand its nature for example, is it merely a product of drug-enhanced suggestibility (Carhart-Harris et al., 2015) for example, interacting with expectations or priming, or does it relate to some more substantive action on brain dynamics for example, shifting them into an entropic or critical regime conducive to regeneration and healing (Atasoy et al., 2017; Carhart-Harris et al., 2014; Schartner et al., 2017; Singleton et al., 2022; Toker et al., 2022; Varley et al., 2020) – see also Carhart-Harris et al. (2022) and the notion of ‘neural annealing’.

Briefly, the hypothesised therapeutic mechanism is that via a well-established (Timmermann et al., 2019) psychedelic-induced increase in the entropy of spontaneous brain activity, overly weighted or ‘canalised’ sub-states (e.g. encoding pathological habits of mind or behaviour) become de-weighted or ‘relaxed’, consistent with an increase in functional plasticity in the brain and mind (Carhart-Harris et al., 2022). This acute ‘entropic brain’ effect may have sub-acute consequences; specifically, it may leave a carryover effect consistent with greater flexibility or dynamism within the global system – that is, brain dynamics and associated cognition, affect and behaviour may become ‘deroutinized’ in a manner consistent with and conducive to improved mental health.

Here it is relevant to invoke a recent work on a generalised or ‘meta’ plastic action of psychedelics – for example, see Temperature or Entropy Mediated Plasticity (TEMP) (Carhart-Harris et al., 2022) and critical period plasticity (Nardou et al., 2023). Relatedly, we may ask whether there is a link between the inner healer experience, an entropic brain action (Carhart-Harris, 2018b), and the regeneration of stress-atrophied synaptic connections (De Gregorio et al., 2022; Moda-Sava et al., 2019) – see also Carhart-Harris et al. (2022) and Nardou et al. (2023). Does the dysregulation of statistical regularities in spontaneous brain activity described by the ‘entropic brain’ action of psychedelics carry over into recalibrated dynamics within the brain and mind – for example, as described here (Carhart-Harris, 2018b). This landscape opening or flattening could be viewed as commensurate with a ‘Bayesian model reduction’ process (Carhart-Harris and Friston, 2019) that is ‘felt’ as an entelechy towards wholeness. Sensations or feelings of apparent psychological insight may accompany these changes in system dynamics. That is, as the brain and mind dynamics shift towards a broader, more open mode of functioning, the natural experiential accompaniment to this is a feeling of embodying a broader perspective on one’s self and past, and relations to others and the world more generally for example, see Peill et al. (2022) and Carhart-Harris et al. (2018b). Insight may be felt in two non-mutually exclusive phenomena: (1) as a broadening or opening and (2) as a data or information decompression, where ordinarily occluded, suppressed or compressed information arises into conscious awareness.

We are still some way from fully understanding whether and how various extrapharmacological components such as external factors, in addition to internal processes, shape psychedelic experiences and subsequent outcomes (Carhart-Harris et al., 2018c). Psychological support appears to have an important influence on clinical outcomes, as evidenced by associations between therapeutic alliances and antidepressant effects (Murphy et al., 2022; Timmermann et al., 2022). Music – otherwise known as the ‘hidden therapist’ in psychedelic therapy (Kaelen et al., 2018) – appears to influence therapeutic outcomes. Experiential and epistemic processes such as emotional release (Roseman et al., 2019) and psychological insight (Davis et al., 2021; Peill et al., 2022) appear to play an important role – and these intuitions are supported by our modelling work here, showing strong covariance between the inner healer effect and emotional breakthrough and mystical-type experiences. Intuitively, all these factors bear relevance to the inner healer phenomenon and speak to a synergistic confluence between an intrinsic biological action and external contextual forces. Future work is required to test hypotheses regarding this putative synergistic interaction (Carhart-Harris et al., 2018c). It may transpire, for example, that the putative intrinsic healing action of psychedelics is not inexorable but rather can be overridden by negative conditions or contexts.

It has been postulated that psychological healing processes may entail a period of suffering/pain, whilst an individual or group faces – and potentially overcomes – existential struggle (Campbell, 2008). The notion of passing through struggle *enroute* to healing is consistent with the so-called ‘healing crisis’ or Jarisch-Herxheimer reaction (Lloyd, 1945) as well as the anthropological and psychological notion of the ‘hero’s journey’ that is, a universally shared human theme of (1) endeavour and adventure, (2) struggle and crisis, followed by (3) triumph or ‘rebirth’. A process that is hypothesised to be circular rather than unidirectional and culturally transcended.

All the above-listed themes are popular in complementary or alternative medicine, recognise potential value in symptom worsening prior to healing. Recent work has suggested that the *early* or onset phase of a psychedelic experience is more likely to be unpleasant or negatively valenced than its latter phases (Brouwer & Carhart-Harris, 2021) and a new conceptual model (R. Carhart-Harris et al., 2022) inspired by recent human functional brain imaging findings (Daws et al., 2022; Singleton et al., 2022) has proposed that the content of psychedelic experiences may gravitate towards psychologically salient themes for mechanistic reasons. More specifically, psychiatric symptoms are hypothesised to be over-reinforced habits of mind or behaviour that, through repetition, become encoded as heavy skews or deformations in an otherwise appropriately well-balanced state space. It is further proposed that such skews occur because the visitation of certain sub-spaces is unconsciously rehearsed or implicitly practiced (e.g. via basal rumination); unlike healthily well-practiced sub-states (such as fine motor skills), they are not carved via a marriage of body, mind/brain and external world; although self-fulfilling thought and behaviour may reinforce unhealthy habits, that is, consistent with so-called ‘active inference’ mechanisms gone awry (Parr et al., 2022).

It is further proposed that such dynamical ‘skews’ that define pathology are hypersensitive to psychedelic drugs’ core entropic action, analogous to surface imperfections being remedied via annealing in metallurgy (R. Carhart-Harris et al., 2022). Future work is required to test whether certain brain effects caused by psychedelics – such as their entropic action (Girn et al., 2023), relating to the so-called inner healer phenomenon and whether the above-described processes are amenable to computational modelling. Such work may help to ground the inner healer phenomenon in explicitly computational and mechanistic terms, aiding a process of construct demystification.

As noted above, exploratory analyses done on request revealed no significant relationships between inner healer scores and challenging experiences. In the naturalistic setting, it appears that the severity of psychological challenge but not its duration is negatively associated with improvements in well-being (Carbonaro et al., 2016). In the clinical setting, it has been difficult to determine the causal influence of a challenging experience. We have previously found some evidence of high-dose challenging experiences deflating otherwise (group average) positive changes in well-being, but this result is likely to be dependent on context (Robin L Carhart-Harris et al., 2018c). A better understanding of the paradox of challenging experiences will greatly aid in disentangling the complex relationship they may have with the inner healer phenomenon as well as mental health change.

Limitations of the present work include the radically brief single-item nature of the measure. Future work should expand on this for example, to examine nuances of the construct, including whether it has a single or multi-factor structure. As pointed out by reviewers, we understand that the inner healer score of 1 (and not 2) drove the relationship with depression outcomes. A larger scale with varied items and a more comprehensive Likert scale/VAS scoring system may help to elucidate this finding. Relatedly, our statistical modelling was kept intentionally simple, and hypothesis driven but future work could deploy more comprehensive regression modelling to better examine interactions between some of the variables discussed above, and the inner healer phenomenon. Another limitation is that we have approached the construct exclusively in its subjective form. Future work examining its neural correlates and whether it can be experimentally manipulated will help explicate its mechanistic nature. One likely effect of this will be to deconstruct and mystify the phenomenon, by revealing the underlying computational and biological processes that are being alluded to via the phenomenology. This process will validate rather than invalidate the phenomenology and should therefore be welcomed.

As with previous constructs often referred to in the psychedelic community but for which there had been little construct validation research (Robin L Carhart-Harris et al., 2018c; Nour et al., 2016), here we have drawn attention to the notion that psychedelics catalyse intrinsic healing processes and offer a first attempt to define and measure the experiential face of this construct to inspire further research on the topic.

## Supplemental Material

sj-docx-1-jop-10.1177_02698811241239206 – Supplemental material for Psychedelics and the ‘inner healer’: Myth or mechanism? Supplemental material, sj-docx-1-jop-10.1177_02698811241239206 for Psychedelics and the ‘inner healer’: Myth or mechanism? by Joseph Peill, Miriam Marguilho, David Erritzoe, Tommaso Barba, Kyle Greenway, Fernando Rosas, Christopher Timmermann and Robin Carhart-Harris in Journal of Psychopharmacology
