# Supplementary material for: Psychedelics and the ‘inner healer’: Myth or mechanism?
Source: J Psychopharmacol. 2024 Apr 12;38(5):417–24. doi: 10.1177/02698811241239206 (PMC11102647; doi:10.1177/02698811241239206)
Supplement: sj-docx-1-jop-10.1177_02698811241239206 – Supplemental material for Psychedelics and the ‘inner healer’: Myth or mechanism? [file sj-docx-1-jop-10.1177_02698811241239206.docx]

Supplementary Material

**Inner healer and acute measures**

As described earlier, for the 25mg condition, correlational analyses revealed a negative correlation between inner healer scores and changes in BDI scores from baseline to two weeks after the 25mg session (r = -.315, p = 0.048). When controlling for EBI, MEQ and CEQ, the negative correlation between inner healer and BDI scores was preserved for EBI (r=-.332, p =0.042) but not for MEQ (r=-.160, p=0.208), CEQ (r=-.312, p=.053). No correlation between inner healer and BDI scores was apparent for the 1 mg condition at the 2-week timepoint (r = -.110, p = 0.586).

|  |  | EBI | MEQ | CEQ | Inner Healer | Inner Healer (controlling for EBI) | Inner Healer (controlling for MEQ) | Inner Healer (controlling for CEQ) |
| --- | --- | --- | --- | --- | --- | --- | --- | --- |
| BDI (2-weeks post-DD1) | Pearson’s r | -.126 | **-.350*** | -.056 | **-.315*** | **-.332*** | -,160 | -.312 |
|  | P value (1-tailed) | .258 | **.032** | .386 | **.048** | **.042** | .208 | .053 |
|  | N | 29 | 29 | 29 | 29 | 29 | 29 | 29 |

**Table 2.** Correlations for 25mg arm EBI/MEQ/CEQ and inner healer vs Beck Depression Inventory (BDI) change from baseline to 2 weeks post dosing day 1 (DD1). The correlation was significant for BDI vs. MEQ (-.350, 1-tailed) but not EBI or CEQ. As showed previously, the correlation was significant for BDI vs. inner healer scores (-.315, 1-tailed) at 2 weeks. When controlling for EBI, the significant correlation was maintained (-.332, 1-tailed) but not for MEQ or CEQ. *Correlation is significant at below 0.05.

For the 25mg condition, correlational analyses revealed a positive correlation between inner healer and emotional breakthrough (r=.749, p<0.001) and mystical experiences (r=.548, p=0.01) but not challenging experiences (r=.074, p=.349) or a general altered state of consciousness (see supplementary material for this).

|  |  | EBI | MEQ | CEQ |
| --- | --- | --- | --- | --- |
| Inner Healer | Pearson’s r | .749*** | .548** | .074 |
|  | P value (1-tailed) | <.001 | <.001 | .349 |
|  | N | 30 | 30 | 30 |

**Table 3.** Correlations for inner healer scores and EBI, MEQ and CEQ. The correlation was significant (positive) for inner healer vs. EBI (.749, 1-tailed) and MEQ (.569, 1-tailed) but not CEQ (.074). **Correlation is significant at the 0.01 level, **correlation is significant at the 0.001 level. Emotional Breakthrough Inventory (EBI), Mystical Experience Questionnaire (MEQ) and Challenging Experience Questionnaire (CEQ).

For the 25mg condition, correlational analyses revealed no correlation between inner healer and acute measures of general altered state of consciousness.

|  |  | Experience of unity | Spiritual Experience | Blissful state | Insightfulness | Disembodiment | Impaired control and cognition | Anxiety | Complex imagery | Elementary Imagery | Audio Visual Synaesthesia | Changed Meaning of percepts |
| --- | --- | --- | --- | --- | --- | --- | --- | --- | --- | --- | --- | --- |
| Inner Healer | Pearson’s r | .235 | .322 | .294 | .276 | .114 | .024 | .285 | .146 | .074 | .034 | .201 |
|  | P value (1-tailed) | .211 | .082 | .114 | .140 | .548 | .901 | .126 | .440 | .696 | .859 | .286 |
|  | N | 30 | 30 | 30 | 30 | 30 | 30 | 30 | 30 | 30 | 30 | 30 |

**Table 4**. Correlations for 25mg arm scores (inner healer) and acute measures (ASC-11D). The correlation was not significant for inner healer vs. all ASC subscales. Altered states of Consciousness – 11 Dimensions (ASC-11D).
